# Classification-based motion analysis of single-molecule trajectories using DiffusionLab

**DOI:** 10.1038/s41598-022-13446-0

**Published:** 2022-06-10

**Authors:** J. J. Erik Maris, Freddy T. Rabouw, Bert M. Weckhuysen, Florian Meirer

**Affiliations:** 1 Inorganic Chemistry and Catalysis, Debye Institute for Nanomaterials Science, Utrecht University, 3584 CG Utrecht, The Netherlands; 2 Soft Condensed Matter and Biophysics, Debye Institute for Nanomaterials Science, Utrecht University, 3584 CC Utrecht, The Netherlands

**Keywords:** Analytical chemistry, Catalysis, Materials chemistry, Software, Software, Molecular imaging, Fluorescence imaging, Imaging techniques

## Abstract

Single-particle tracking is a powerful approach to study the motion of individual molecules and particles. It can uncover heterogeneities that are invisible to ensemble techniques, which places it uniquely among techniques to study mass transport. Analysis of the trajectories obtained with single-particle tracking in inorganic porous hosts is often challenging, because trajectories are short and/or motion is heterogeneous. We present the DiffusionLab software package for motion analysis of such challenging data sets. Trajectories are first classified into populations with similar characteristics to which the motion analysis is tailored in a second step. DiffusionLab provides tools to classify trajectories based on the motion type either with machine learning or manually. It also offers quantitative mean squared displacement analysis of the trajectories. The software can compute the diffusion constant for an individual trajectory if it is sufficiently long, or the average diffusion constant for multiple shorter trajectories. We demonstrate the DiffusionLab approach via the analysis of a simulated data set with motion types frequently observed in inorganic porous hosts, such as zeolites. The software package with graphical user interface and its documentation are freely available.

## Introduction

Mass transport is widely studied across the natural sciences: from the movement of a reactants into a catalyst particle to cellular transport driven by motor proteins^1,2^. To understand and model transport phenomena and predict associated properties, quantitative experimental input is crucial. An excellent way to obtain this is to record the location of a single moving object, such as a molecule or nanoparticle, as a function of time, yielding a so-called trajectory. Already back in the 1900’s, Perrin studied the movement of single granular and colloidal particles by recording their trajectory, and by doing so, was the first to experimentally verify Einstein’s work on Brownian motion^3,4^. The modern era of trajectory-based analysis started with the development of algorithms to localize and track particles from time-lapse microscopy videos^5,6^. These methods together with fluorescence microscopy techniques, such as single-molecule localization microscopy, enable tracking of single fluorescing molecules, quantum dots, and colloids with nanometre resolution^1,2,6,7^.

Molecules with the same chemical identity can display very different motion behaviour as a result of the complex environment where the diffusion takes place. Driven by the notion that ensemble-averaging obscures heterogeneities and thus hides important features for cellular function, single-particle tracking has had a crucial role in the discovery of cellular organisation and a variety of cellular processes^8^. In the field of materials science, different motion behaviours have been observed for fluorescent molecules diffusing through a porous catalyst particle^1^. Interpretation of a data set with fluorophores displaying different motion behaviours requires sufficient statistics on the occurrence of the motion behaviours and a quantitative description of each motion behaviour via a diffusion model. This is challenging as the measured trajectories are often short, i.e. ~5–15 frames, as a result of fast diffusion, rapid photobleaching, and blinking of the fluorophores, and individually do not contain sufficient information for a reliable quantification of the motion.

We present in this work an open access, freely available software package “DiffusionLab” to perform quantitative analysis of datasets of single-molecule trajectories. DiffusionLab is versatile, is user-friendly, and can be readily used to perform complex tasks without the need for programming experience. Most other software packages focus on the quantification of multiple normal diffusion states directly from the analysis of the displacements, using various methods such as fitting of the cumulative distribution of squared displacements^9–15^. In contrast to these software packages, DiffusionLab first simplifies the data set by classification of the trajectories into smaller populations with similar motion behaviour prior to diffusion quantification. In this way, motion heterogeneity can be visualized and quantified in a robust way that is not only dependent on fitting of a single (multistate) diffusion model to the displacements^1,16^. Consequently, the DiffusionLab software can handle trajectory data sets containing a mixture of motion types such as normal, confined, and directed diffusion by treating them separately. DiffusionLab focusses on robustness and ease of use to make the tools for quantitative analysis of complex trajectory data sets broadly available to the scientific community.

The combination of trajectory classification and motion analysis has been proven to be a powerful approach for the analysis of complex data sets with short trajectories, particularly in the field of materials science as evidenced by work from our group using the DiffusionLab software^1,16^. The potential of feature-based classification has also been reported by Lerner et al. for the classification of confinement in trajectories mixed with normal or directed diffusion^17^. Lerner et al. classified the trajectories based on three features that described properties of individual trajectories and, subsequently, quantified confinement within the classified trajectories with mean squared displacement analysis using the “msdanalyzer” software^18^. DiffusionLab has a wide range of built-in trajectory features (or “properties”) as well as the option to add custom features by the user to provide descriptors for many different motion types. The classification is done using these features, either by setting thresholds manually or with feature-based machine learning methods on a user-generated training set. Recently, machine learning methods have been developed for the detection and classification of the different diffusion types, which are mostly focussed on the recognition of anomalous diffusion^19–25^. In contrast to the approach employed in DiffusionLab, these models are trained by simulated trajectories with a known set of diffusion models. The presence of anomalous diffusion is some cases not clear from mean squared displacement analysis and can be confused with normal diffusion^8,26^. Further classification by state-of-the-art machine learning models after classification in DiffusionLab could be required to identify the type of anomalous diffusion^27^.

Single-molecule localization microscopy allows the localisation of fluorophores with a spatial resolution much smaller than the diffraction limit of light^7^. When light from a point source passes through a lens, it spreads out before it reaches the camera due to diffraction of the relayed light. The “point spread function” (PSF) is a function that describes how a point source at position *r* in the sample makes an image at position *r’* on the camera. The PSF stretches over all positions *r’* on the camera but is virtually zero apart from a small region. If the pixel size is sufficiently small, a mathematical description of the PSF can be fit to the camera image *r’* and the position of the point emitter *r* is retrieved with nanometre precision. A Gaussian function with a full width at half maximum in the order of the emission wavelength is often used to describe the PSF. Since the fluorophores are generally very small, the PSF is virtually the same for light emitted anywhere from the fluorophore’s volume and they can be regarded as point sources. If the fluorophore density in a single micrograph is sufficiently low that the non-zero regions of the PSFs do not overlap, the location coordinates of each fluorophore can be determined rapidly with available algorithms^28,29^. In the next step, the localisation coordinates from a time series that most likely belongs to the same molecule are grouped together into a trajectory. Grouping coordinates into trajectories is often achieved by an algorithm that minimizes a cost function, which depends on e.g. the displacement between consecutive coordinates^7,8,28–31^. The DiffusionLab software imports trajectories from third-party localization and tracking applications and provides the tools to analyse the trajectories via classification and motion analysis.

Mean-squared displacement (MSD) analysis is one of the most common tools to quantify the motion of a fluorophore described by a trajectory, because of its visual interpretability^32,33^. The shape of the MSD curve is dependent on the motion behaviour of the diffusing particle. While normal (Brownian) diffusion is characterised by a linear relation between the MSD and the delay time $t_{n}$, confined motion results in an MSD that reaches a plateau for long delay times and directed motion can be identified by a parabolic MSD curve. Anomalous diffusion can in some cases be mistaken for normal diffusion in the MSD analysis, and careful interpretation is required in systems where anomalous diffusion is expected. The MSD is computed from a time series of positions ${\varvec{x}}_{0} ,{\varvec{x}}_{1} , \ldots ,{\varvec{x}}_{N}$ for a single trajectory as

1 $${\text{T-MSD}}\left( {t_{n} } \right) = \frac{1}{N - n + 1}\mathop \sum \limits_{i = 0}^{N - n} \left| {{\varvec{x}}_{i + n} - {\varvec{x}}_{i} } \right|^{2}$$

with the delay time $t_{n} = n{\Delta }t$ for $n = 1, 2,...,N$ and the time between frames ${\Delta }t$^7,32–35^. We will refer to this MSD as the time-averaged MSD (T-MSD) to distinguish it from the time–ensemble averaged MSD discussed later in the text—the term MSD will be used when this distinction is not required. In practice, the MSD curve will be affected by noise on the coordinates ${\varvec{x}}_{i}$ determined experimentally, because of the localisation error due to photon-counting noise and image blur as a result of motion within the exposure time of a microscope frame. For free two-dimensional normal diffusion including localisation error and motion blur, the measured mean squared displacement ${\text{MSD}}\left( {t_{n} } \right)$ is described by^32,34^,

2 $${\text{MSD}}\left( {t_{n} } \right) = 4Dt_{n} + 4\left( {\sigma^{2} - 2RD{\Delta }t} \right){\text{ for }}n \ge 1$$

with $D$ the diffusion coefficient, $\sigma$ the localization error, and $R$ the motion blur coefficient. The first term in Eq. (2) describes how the MSD increases as function of delay time due to normal diffusion, while the second term adds a constant factor accounting for localization error and motion blur, which are both errors introduced by the experimental measurement. $R$ can take values in the interval $\left[ {0, 1/4} \right]$ and is dependent on the detection scheme. In the case of no motion blur, $R = 0$, while $R = 1/6$ if the exposure time of the camera equals the time between frames. Ideally one would fit (a set of) trajectories that can be described by a single diffusion constant; however, if the individual fluorophores switch between diffusive states, the MSD analysis will report only an average diffusion constant.

We include (T-)MSD curve analysis in DiffusionLab as primary motion estimator because it is accessible, well known across the scientific community, and robust for many different motion behaviours. The caveat of T-MSD analysis is that the fitted parameters can be biased when the trajectories are short (Sec. S1.1)^8,34^. This is particularly a problem for single-molecule trajectories recorded in inorganic porous hosts, which are generally short and have heterogeneous underlying motion behaviour. Bias in the T-MSD curve makes the identification of the motion behaviour ambiguous and introduces bias in the fitted parameters. Unfortunately, these trajectory properties are inherent to the experiment and cannot be changed substantially—even by experiment design^7^. Therefore, we classify the individual trajectories based on similarity and pool them to get their population average, which is a well-known concept in processing hyperspectral images^36^. With this approach we reduce noise and bias in the population-averaged MSD curve, and as a result we obtain the motion type and fitted parameters of the pooled trajectories with improved performance. Ultimately, it allows the spatial mapping of motion heterogeneity on level of single short trajectories.

In this work, we explain the methodology of DiffusionLab and demonstrate it via the classification of trajectories of a simulated example data set. This example contains trajectories of fluorophores that show transient trapping, which is common for fluorophores diffusing in a nanoporous host^1,7,16,37,38^. We demonstrate how to perform classification via machine learning. Finally, the motion of the classified populations is analysed and quantified with MSD analysis.

## Methods

We simulated synthetic trajectories of emitters exhibiting transient trapping. The details are given in the Sec. S1.2. Briefly, a synthetic dataset was generated in three steps: (1) simulation of the coordinates of 3-dimensional *xyzt* trajectories^39^; (2) generation of synthetic *xyt* time lapse video frames from 3-dimensional trajectories using a simulated point spread function^40,41^ and homogeneous background signal; and (3) localization and tracking of the generated synthetic time lapse video with conventional fitting routines^29^. To simulate transient trapping, the coordinates of a random walk with two diffusion states was simulated in step (1). The diffusion constant of the first diffusion state was set to zero (${D}_{1} = 0$) while the other was set to ${D}_{2}=1.0\times {10}^{-12}$ m^2^ s^−1^. The probability to change diffusion state was 0.02 per frame (0.4 s^−1^). A fixed number of trajectories was simulated in a three-dimensional box with periodic boundary conditions during the full simulation time. Every trajectory coordinate in (1) resulted in a PSF “placed” in the time-lapse video (2). To simulate motion blur, the trajectory coordinates in step (1) were computed with a five times higher time resolution than the synthetic time lapse video in (2). This means that we placed five PSFs along the path the emitter has travelled during one frame. Photon-counting noise and camera noise were simulated in step (2) resulting in an imprecision in the localization in (3), which altogether introduced a localisation error. In the final step, localisation and tracking were done with the DoM plugin (Detection of Molecules, https://github.com/ekatrukha/DoM_Utrecht) for ImageJ^29^. A small fraction of the simulated emitters was in the microscope frame, i.e., focal volume, at the same time. Importantly, the emitter was only tracked by the DoM plugin when it was sufficiently in focus. Out-of-focus emitters were too broad or had insufficient counts to be picked up by the tracking algorithm. As a result, the three-dimensional trajectories were split into many shorter trajectories due to in- and out-of-focus movement of the simulated emitters.

The hierarchical classification tree was constructed from a manually classified training set of 100 trajectories. The training set was used to automatically select the threshold and the trajectory properties, i.e., features. We allowed all trajectory properties available in DiffusionLab to be used by the model, which was the “elongation”, “elongation angle”, “entropy”, “length”, “minimum bounding circle radius”, “minimum bounding circle minus the centre of mass”, “number of points”, and “tortuosity” (see also Documentation Section 3.1.1). The maximum number of splits in the tree was limited to five in all examples, because a larger tree did not lead to better classification and could cause overfitting.

The classification and analysis of the simulated trajectories was done with version 1.1.0 of the DiffusionLab software package. The MATLAB installer and source code are available online: https://github.com/ErikMaris/DiffusionLab. The latest version of the documentation can be downloaded via https://diffusionlab.readthedocs.io/_/downloads/en/latest/pdf/.

## Results and discussion

### The workflow and concepts of DiffusionLab

**Figure 1:**
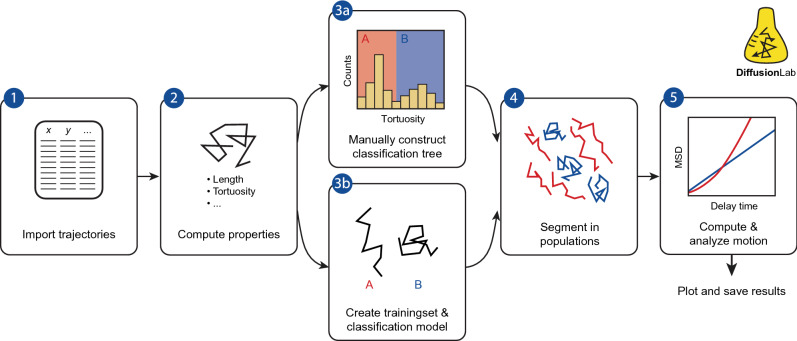
Workflow of population-based motion analysis in DiffusionLab: (1) the trajectories are imported; (2) a set of descriptors (properties) of each trajectory is computed; (3) a classification model is constructed, this can either be done manually as a hierarchical classification tree (3a) or via supervised machine learning (3b); (4) the classification model is used to classify all trajectories in populations with similar motion behaviour; and (5) the mean squared displacement (MSD) of the populations is analysed separately. Finally, the properties and motion analysis results can be plotted, saved and/or exported.

The workflow of the classification-based motion analysis in DiffusionLab is given in Fig. 1. In the first step, the trajectories are imported from a tracking software. At the time of writing, DiffusionLab supports the import of trajectories from the software package Localizer^28^ and the ImageJ plug-in DoM^29^. Moreover, the import of simulated trajectories from COMSOL Multiphysics® is supported to compare experimental and simulated data sets^42^. Step-by-step instructions for how to export the trajectories from the respective software are given in the Documentation section 2.2. In the second step, a set of descriptors of each trajectory is computed, which are scalars that capture a property of the trajectory. Examples of these properties are the length and tortuosity of the trajectory. Both two and three-dimensional trajectories are supported; however, not all properties are defined in three dimensions. A description and physical interpretation of the properties is given in the Documentation section 3.1.1.

**Figure 2:**
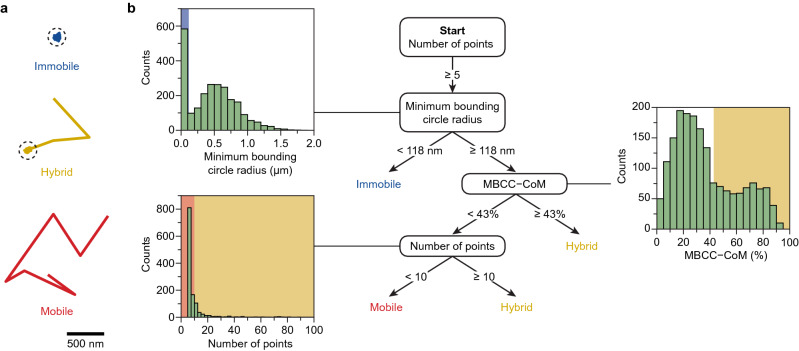
(**a**) Example trajectories of the immobile, hybrid, and mobile population. The dashed circle has a radius of the minimum bounding circle radius and marks the immobile segments in the trajectories. (**b**) Classification tree obtained with machine learning on a training set of 100 manually classified trajectories for a simulated dataset with transient trapping. The classification tree is read from top to bottom for each trajectory, weighing one property at each branch, until the trajectory is classified as either mobile, immobile, or hybrid. At each split, the histogram of the property is given for all trajectories of the branch, with the background colour indicating the threshold value of the split.

A classification model is constructed to classify the trajectories into populations with similar motion behaviour. The populations are identified by the user and classification is done via a hierarchical classification tree. The hierarchical classification tree is read from top to bottom for each trajectory, weighing one property at each branch, until the trajectory is classified (example in Fig. 2). Hierarchical classification trees have the advantage that they are easily interpretable and important properties and their thresholds are readily available from the model^43^. This aids rational design of the classification model when constructed manually. For machine learning, the classification tree model has the advantage that it is very well interpretable compared to other machine learning models. In DiffusionLab, hierarchical classification trees can be constructed manually or via supervised machine learning. For manual construction, DiffusionLab has various tools to find important properties for classification and to set the thresholds. The biplot shows the properties with a high dispersion that are therefore good candidates to implement in the tree (see Sec. S1.3). Histograms and correlation plots can be generated to find thresholds in e.g., bimodal distributions as is shown in Fig. 1, step 3a. These thresholds can be optimized by applying the classification and visually assessing the result. In supervised machine learning, the model maps an input (trajectory properties) to an output (motion behaviour type) based on example input–output pairs. The example pairs are called a “training set”, which are here a set of representative trajectories labelled with their motion behaviour. DiffusionLab offers a tool to manually classify a subset of experimental trajectories to obtain such a training set. This is then used to compute a hierarchical classification tree model. On top of that, MATLAB’s Classification Learner app can be used to construct a classification model with any classification models available in MATLAB, which includes support vector machines and K-nearest neighbours. In the fourth step, the classification model is used to classify all trajectories in populations.

The motion of the classified populations is analysed in the last step (Fig. 1, step 5), and this is where the power of the classification-based approach becomes truly visible. With DiffusionLab, the user can quantify both the motion of individual trajectories and the ensemble behaviour of each population. Analysis of the individual trajectories reveals the heterogeneity in molecular motion on the single-molecule level. However, in data sets with short trajectories, the statistical relevance of a single trajectory is limited. Because the trajectories have been classified into populations with similar motion behaviour, we can overcome this by analysing the ensemble-averaged behaviour and diffusion parameters. T-MSD analysis is known to suffer from bias in the estimated diffusion parameters due to correlation in the displacements^8,34^. Since the detected motion behaviour is dependent on the relation between the MSD and the delay time, the bias makes it particularly challenging to determine the diffusion model that best characterizes the motion of the emitters^8^. The time–ensemble averaged MSD (TE-MSD) averages the T-MSD over a subset of trajectories, thereby averaging over any correlations within the trajectories

3 $${\text{TE-MSD}}\left( {t_{n} } \right) = \frac{1}{{J_{n} }}\mathop \sum \limits_{{j,N_{j} \ge n}} \frac{1}{{N_{j} - n + 1}}\mathop \sum \limits_{i = 0}^{{N_{j} - n}} \left| {{\varvec{x}}_{j,i + n} - {\varvec{x}}_{j,i} } \right|^{2}$$

with the time series of positions ${\varvec{x}}_{j,0} ,{\varvec{x}}_{j,1} ,...,{\varvec{x}}_{{j,N_{j} }}$ for trajectory $j$. For each delay time $t_{n}$, we include an ensemble of $J_{n}$ trajectories with $N_{j} \ge n$. The bias in the TE-MSD is strongly reduced with respect to the T-MSD, which results in a interpretable relation between the MSD and the delay time over a longer time domain, facilitating the determination of the motion behaviour type^8^. Moreover, the diffusion constant can be readily extracted from the TE-MSD, which allows direct comparison within and between different experiments. We use the T-MSD for the analysis of individual trajectories, while TE-MSD is used to analyse a population of trajectories. Altogether, using these two complementary approaches we obtain both insight in the motion heterogeneity on the single-molecule level as well as the mean diffusion parameters.

### A simulated example of trajectory classification and motion analysis

We demonstrate the workflow of DiffusionLab on a dataset of simulated trajectories (see Methods). The trajectories were simulated from random walkers in three dimensions that could switch between an immobile (${D}_{1}=0$ m^2^ s^−1^) and mobile state (${D}_{2}=1.0\times {10}^{-12}$ m^2^ s^−1^), mimicking transient trapping often observed in porous solids^1,7,16,37,38^. We imported the data set into DiffusionLab and find 10,600 individual trajectories (Fig. 1, step 1). Before computing the properties in step 2, we removed trajectories with fewer than five localizations^17^. In this way, we reject trajectories and unconnected localisations whose properties, such as a minimum bounding circle radius, are not or ill-defined. Moreover, the localisation and tracking algorithm sometimes finds localisations due to fluctuations in the camera noise. Since these erroneous localisations are random, the probability that they form a trajectory becomes smaller for longer trajectories. Thus, we can eliminate most of these from the analysis by removing trajectories with fewer than five localizations. Finally, for a trajectory of five localisations, the minimum number of points in the MSD curve is four, which we consider the minimum for MSD analysis. Removing the trajectories with less than five localizations reduces the number of trajectories to 2433. DiffusionLab then computes and stores the trajectory properties, as explained in the Documentation Section 3.1.1.

Next, the hierarchical classification tree was created (Fig. 1, step 3), and we first identified the motion types present in the data set. Although the movement of all emitters was simulated using the same underlying diffusion constants, we observed three qualitatively different types of trajectories, in agreement with the experimental results of Hendriks and Meirer et al*.*^1^ (Fig. 2a and Sec. S1.4). For some trajectories, the coordinates are separated from each other by no more than the localisation error. These do likely not reflect actual movement, but the apparent motion is due to the localisation error. We call these trajectories ‘immobile’. Other trajectories show continuous movement without interruptions, which we label ‘mobile’ trajectories. A third group of ‘hybrid’ trajectories has both mobile and immobile periods. Next, we manually classified a hundred trajectories, assigning each to one of the three motion types, and used this as a training set for machine learning. A training set of just a hundred trajectories is typically considered to be very small for machine learning; nevertheless, we show that this can be sufficient to construct a classification tree with reasonable accuracy. The classification tree has a resubstitution loss of 2%, which means that two of the hundred trajectories in our training set were incorrectly classified by the tree. With a small training set as is discussed here, we highly recommend to assess the tree’s performance via the resubstitution loss as well as by visual inspection of the complete data set after classification. The classification tree obtained is shown in Fig. 2b. A discussion of the performance of other estimators can be found in Section S1.5. Reproduction of the motion analysis with segmentation using a different training set yields a very similar tree and classification results (Sec. S1.6). Interestingly, the same track properties, that is, the minimum bounding circle radius (MBCR), the minimum bounding circle centre minus the centre of mass (MBCC–CoM), and the number of points, were again found to be most important as previously reported in the classification tree of Hendriks and Meirer et al*.*^1^. The tree in their work was constructed with machine learning from a training set with mobile, hybrid, and immobile trajectories obtained from an experimental dataset of molecules diffusing in a porous catalyst particle. At every split of our classification tree, the histogram of the respective trajectory property is given in Fig. 2b. A bimodal distribution is clearly visible in the histogram of the MBCR and MBCC–CoM. The machine learning algorithm had found good threshold values to split the bimodal distribution without having access to the complete data set (shown in Fig. 2b). The stochastic nature of the random walk results in some overlap between the properties of the various motion types, as can been seen in the histogram, and the classification is successful if a vast majority of the trajectories in a population have been correctly classified.

We can rationalize why the MBCR, MBCC–CoM, and number of points are good properties to classify a data set with transient trapping. It is no surprise that the MBCR is a good property to classify immobile trajectories, because the emitter does not move, and localisations are scattered around the emitter’s true position. In Section S1.7, we calculate the probability that an immobile trajectory falls entirely within a minimum bounding circle with some threshold radius. A threshold value of the MBCR of 118 nm is reasonable compared to the localization errors of 12 and 28 nm of an in-focus and 400 nm out-of-focus fluorophore^44,45^. The probability that a trajectory with 17 localisations, which is the mean length for the immobile population, fits within the minimum bounding circle is > 99.99% for an in-focus immobile emitter, while it drops to 99.95% for an out-of-focus one. Thus, the value of the MBCR allows for out-of-focus emitters to be classified as immobile, while keeping it as low as possible to not include many hybrid trajectories. The MBCC–CoM is a good property to distinguish hybrid trajectories from mobile ones. Because it describes how far the centre of the minimum bounding circle and the centre of mass are apart, its value will be higher when the trajectory has a spatially asymmetric distribution of localisations. This is a characteristic of a trajectory with an immobile segment followed by a mobile segment, in other words: a hybrid trajectory. The number of points is a good indicator for whether the trajectory is either hybrid or mobile. Mobile trajectories are a result of emitters rapidly moving in- and out-of-focus resulting in short trajectories, while the immobile segments in hybrid trajectories make the trajectory longer. Because these properties are good descriptors for this type of motion behaviour, we demonstrated that the presented classification tree can be manually adapted to classify a data set with transient confinement (Sec. S1.8). Next, we used the obtained classification tree to classify all trajectories in the transient trapping data set (Fig. 1, step 4).

**Figure 3:**
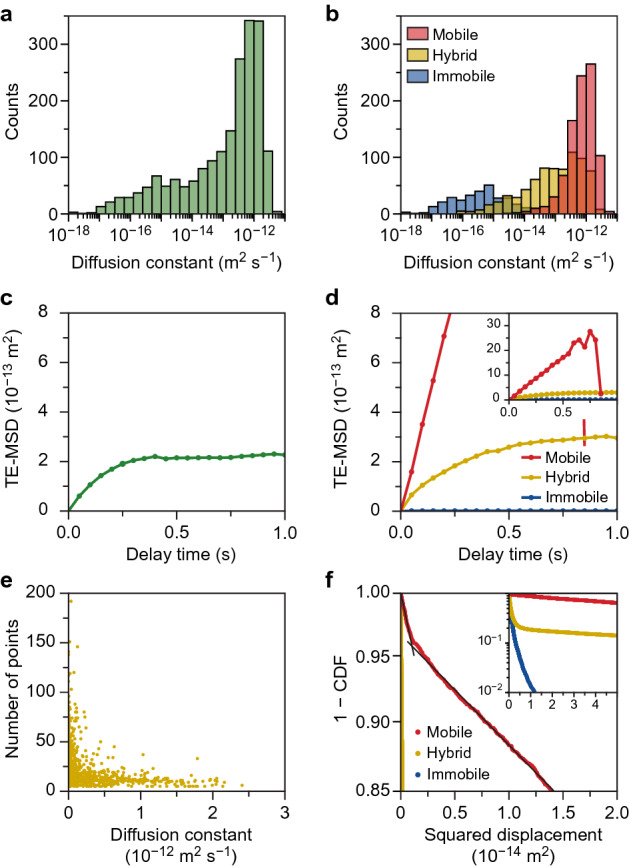
(**a**,**b**) Histogram of the measured diffusion constant as obtained by time-averaged mean squared displacement (T-MSD) analysis per trajectory (**a**) before and (**b**) after classification. Negative measured diffusion constants are not displayed in the logarithmic scale. (**c**,**d**) Zoom of the time–ensemble averaged mean squared displacement (TE-MSD) (**c**) before and (**d**) after classification. A zoom-out is shown in the inset in (**d**). (**e**) Correlation plot of the diffusion constant and number of points of the hybrid population. Each dot represents a single trajectory. (**f**) One minus the cumulative distribution function (CDF) of squared displacements for a delay time of one frame of all trajectories plotted with a logarithmic *y*-axis. The colours indicate the populations after classification. The solid black lines are a guide to the eye to indicate two regimes in the cumulative density probability of the mobile trajectories. A zoom-out is shown in the inset.

The motion of the different populations is quantified with MSD analysis of individual trajectories and in ensemble (Fig. 1, step 5). The diffusion constant was measured from a linear least-squares fit of the T-MSD curve including 25% of the shortest delay times and at least three points in the fit. A histogram of the measured diffusion constants of individual trajectories is given in Fig. 3a,b. Prior to classification, we find a continuous range of diffusion constants over six orders of magnitude (Fig. 3a). The distribution contains all information about the heterogeneity of mobility and motion behaviour, but it is hard to interpret. By pooling the data into motion behaviour classes, we learn how the mobility and motion behaviour are related and how they change per experiment. To understand the origin of this wide range in diffusion constants, we consider the same histogram after classification (Fig. 3b). Remarkably, the immobile trajectories make up for most of the spread, while we would expect the diffusion constant of immobile emitters to be $D = D_{1} = 0$. Localisation noise dominates the measured diffusion constants of these immobile emitters, and the spread does not reflect a true distribution of diffusion constants. As expected, the immobile trajectories have a diffusion constant close to zero. The distribution of the diffusion constant of mobile trajectories is narrower and centred around $D = D_{2} = 10^{ - 12}$ m^2^ s^−1^, i.e., the true mobility of the mobile diffusion state in the simulation. The hybrid trajectories have a wider range of diffusion constants in between those of the immobile and mobile diffusion states, because of the varying length of mobile and immobile periods in the trajectories.

The TE-MSD curve provides insight in the mean motion behavior of the emitters via the shape of the curve and fit of an appropriate diffusion model. We compare the TE-MSD before and after classification (Fig. 3c,d). The shape of the TE-MSD curve prior to classification in Fig. 3c resembles confined motion, marked by the plateau at long delay times^39^. However, we do not expect confined motion in this data set, since the transient trapping diffusion model does not impose confinement on the emitters. Interestingly, we find the same plateau after classification in the hybrid trajectories’ TE-MSD curve, while the mobile and immobile TE-MSD curves are a straight line (Fig. 3d). To explain the plateau at long delay times, we first consider the TE-MSD curve of the hybrid trajectories. The correlation plot of the diffusion constant and the number of localizations of the hybrid trajectories shows that trajectories with few localizations have a high diffusion constant and vice versa (Fig. 3e). The number of localisations in the trajectory is highly dependent on the number of immobile segments, because the emitter cannot move out of focus during these immobile periods. At long delay times in the TE-MSD curve, only the trajectories with many localizations (thus a longer time in the immobile state) contribute, which results in a decrease in the slope of the curve. Now that we understand the origin of the plateau at long delay times in the TE-MSD of the hybrid trajectories (Fig. 3e), we can explain the TE-MSD prior to classification (Fig. 3d). Here, the curve also flattens to a plateau value—even more rapidly than for the hybrid trajectories—because short mobile trajectories and long immobile trajectories contribute to the TE-MSD curve as well. In contrast to the hybrid trajectories, the TE-MSD curves of the mobile and immobile trajectories are a straight line. This confirms that the mobile trajectories are due to normal diffusion. The slope of the TE-MSD of the immobile trajectories is zero, i.e., the apparent diffusion constant is zero, which is in line with their immobile motion behaviour.

The diffusion constant of the mobile trajectories is extracted from the TE-MSD curves using the model for normal Brownian diffusion. For our data set, we found *D* = 9.23 ± 0.14 × 10^–13^ m^2^ s^−1^. This value is slightly but statistically significantly lower than the true diffusion constant of the mobile state of *D* = 1.0 × 10^–12^ m^2^ s^−1^ that we had put in our simulations. This is attributed to a small number of immobile steps in the mobile trajectories, which reduces the measured diffusion constant of the population. We can estimate the fraction of immobile steps in the mobile population with the cumulative distribution function (CDF) of squared displacements for a delay time of one frame (Fig. 3f). Normal diffusion in two dimensions should yield^46^,

4 $$1 - {\text{CDF}}\left( {r^{2} ,t_{n} } \right) = {\text{exp}}\left( {\frac{{ - r^{2} }}{{{\text{MSD}}\left( {t_{n} } \right)}}} \right)$$

with $r^{2}$ the squared displacement at a delay time $t_{n}$. This produces a straight line in a semi-log plot of 1—CDF against $r^{2}$. Multiple decays in the 1—CDF curve indicate that multiple populations of emitters with a different diffusion constant are present, each with a different MSD resulting in a different slope of the curve. For the mobile trajectories, we find a transition from a fast decay (immobile displacements) to a slower decay (mobile displacements) (see black guides to the eye in Fig. 3f). This confirms that ~4% of the displacements in mobile trajectories are immobile, which mainly explains a slight underestimation of the true simulated diffusion constant in the TE-MSD analysis. It also shows that the classification is not perfect, and that motion analysis could be further improved by fitting the 1—CDF curve directly. The remainder of the discrepancy between the measured and true diffusion constant originates from imperfect linking of the localisations into tracks. For the immobile trajectories, we find *D* = 0 ± 4 × 10^–18^ m^2^ s^−1^ confirming that the diffusion constant of the immobile trajectories is zero—as expected.

In summary, we have demonstrated how the DiffusionLab software can be used to analyse a set of trajectories originating from complex heterogeneous motion. The software is a powerful tool for a wide range of materials scientists studying the motion of molecules and nanoparticles in porous materials, including but not limited to zeolite-based materials^1,16^. Adsorption and confinement play a big role in such porous materials, and classification prior to motion analysis can greatly increase interpretability, avoid bias, and allows for more reliable spatial mapping of diffusion heterogeneity. In previous experimental work using the DiffusionLab software, we have demonstrated this approach in a study of molecular diffusion through a porous catalyst particle, which is industrially used to “crack” long organic molecules into smaller—more useful—chemical building blocks^1^. The pore space of the catalyst particle is heterogeneous in material composition and porosity, with pore sizes ranging from the macropore to the micropore regime. The complex structure was reflected in the measured heterogeneous molecular motion behaviour. By classification in mobile, hybrid, and immobile trajectories, diffusivity of only the mobile trajectories could be compared with the bulk diffusion coefficient of feedstock molecules, which turned out to be very similar in magnitude, thereby validating the approach. In other work using DiffusionLab, we have tracked the motion of single oligomers in the pores of ZSM-5 zeolites^16^. By performing classification and motion analysis, we could not only compare the average diffusion constant between experiments, but also explain whether differences were the result of a change in diffusivity during and/or frequency of the mobile periods. This had led to a detailed insight in pore geometry–mass transport relationships on the microscopic scale. These successful applications from materials science recommend the DiffusionLab approach for the field of life sciences as well. The motion types supported by DiffusionLab have been frequently observed in cellular environments. For example, directed motion has been reported when vesicles are transported along microtubules by molecular motors, transient trapping when molecules interact with actin proteins, or confined diffusion when molecules are confined by cell membrane components^2,8^.

## Conclusions and outlook

The DiffusionLab software package, which is open access and freely available to the scientific community, was developed for the analysis and quantification of motion from single-particle trajectories with (1) heterogeneous motion and/or (2) trajectories with only a few localizations. The trajectories are first classified into populations with similar characteristics to which the motion analysis is tailored in a second step. DiffusionLab provides the tools to classify trajectories based on their motion type either with machine learning or manually. MSD analysis is available to perform quantitative motion analysis of the trajectories. The software can compute the diffusion constant for an individual trajectory if it is sufficiently long, or the average diffusion constant for multiple shorter trajectories. We demonstrated DiffusionLab’s workflow with a set of simulated trajectories with a transient trapping model. The data set showed heterogeneous motion behaviour and contained many trajectories with only a few localizations. Using DiffusionLab, we were able to interpret and quantify the motion in this complex data set. Moreover, DiffusionLab has been successfully used to analyse experimental single-molecule trajectories recorded in porous materials as reported previously by our group^1,16^. Future developments of the software will focus on the extension of the motion analysis methods, including a fit of the 1—CDF curves, as well as the addition of new properties for trajectories with many localizations.

## Supplementary Information

Supplementary Information.

## Data Availability

The newest version of the DiffusionLab software is available from the GitHub repository (https://github.com/ErikMaris/DiffusionLab) along with documentation hosted on Read the Docs (https://diffusionlab.readthedocs.io/en/latest/). Both the MATLAB app installer and the source code are available here. The trajectories, training sets, and classification trees used in this work are available from the DiffusionLab repository. The synthetic microscopy movies used to generate the trajectories used in this work as well as the code simulate them will be shared by the lead contact upon reasonable request. The movies were analysed with the DoM plugin (Detection of Molecules, https://github.com/ekatrukha/DoM_Utrecht) for ImageJ^29^.
