# Supplementary material for: Classification-based motion analysis of single-molecule trajectories using DiffusionLab
Source: Sci Rep. 2022 Jun 10;12:9595. doi: 10.1038/s41598-022-13446-0 (PMC9187757; doi:10.1038/s41598-022-13446-0)
Supplement: Supplementary file 1 — Supplementary Information. [file 41598_2022_13446_MOESM1_ESM.docx]

Classification-Based Motion Analysis of Single-Molecule Trajectories Using DiffusionLab

J. J. Erik Maris,^1^ Freddy T. Rabouw,^1,2^ Bert M. Weckhuysen,^1^ Florian Meirer^1,*^

*To whom correspondence should be addressed. Email: f.meirer@uu.nl

^1^Inorganic Chemistry and Catalysis, Debye Institute for Nanomaterials Science, Utrecht University, 3584 CA Utrecht, the Netherlands

^2^Soft Condensed Matter and Biophysics, Debye Institute for Nanomaterials Science, Utrecht University, 3584 CA Utrecht, the Netherlands

Contents

[S1 Supplementary Notes 3](#_Toc101426192)

[S1.1 Correlation in the Mean Squared Displacement of a Single Trajectory 3](#_Toc101426193)

[S1.2 Trajectory Simulation 4](#_Toc101426194)

[S1.3 Find Important Properties for Manual Classification with the Biplot Tool 7](#_Toc101426195)

[S1.4 Trajectory Plot and Artifact Handling 8](#_Toc101426196)

[S1.5 Performance of Classification Models 9](#_Toc101426197)

[S1.6 Robustness of Computation Classification Tree 10](#_Toc101426198)

[S1.7 Probability of an Immobile Trajectory to Fall Within the Minimum Bounding Circle Radius 12](#_Toc101426199)

[S1.8 Classification of Transient Confinement with a Modified Classification Tree 13](#_Toc101426200)

[S2 References 15](#_Toc101426201)

# Supplementary Notes

## Correlation in the Mean Squared Displacement of a Single Trajectory

The mean squared displacement $\text{T-}\text{MSD}(t_{n})$ values (Eq. 1) are highly correlated and statistically dependent when they originate from the same time series of positions $\boldsymbol{x}_{0},\boldsymbol{x}_{1},...,\boldsymbol{x}_{N}$ (Fig. S1a–b). In other words, because of correlation in $\boldsymbol{x}_{i}$, $\boldsymbol{x}_{i+n}$, $\boldsymbol{x}_{i+1+n}$, and $\boldsymbol{x}_{i+1}$, the $\text{T-}\text{MSD}(t_{n})$ computed from the mean of $\left| \boldsymbol{x}_{i+n}-\boldsymbol{x}_{i} \right|^{2}$| and $\left| \boldsymbol{x}_{i+1+n}-\boldsymbol{x}_{i+1} \right|^{2}$ is correlated as well (Fig. S1c). One would expect that by adding more data points to the fit of the T-MSD, i.e., at longer delay times, the quality of the fit would improve. However, the correlation is so strong that when more delay times are included in the fit, the added noise could exceed the added signal leading to a decrease in the quality of the diffusion constant estimate^1^. The precision of the diffusion constant estimate is strongly dependent on the number of delay times that are included in the fit of the T-MSD curve. The optimal number of delay times to include in the fit can be computed and used in the fit of the T-MSD curve^2,3^. However, this is not straightforward when the trajectory has missing positions due to blinking and is unfeasible when the fluorophore switches between diffusive states. Therefore, we recommend to fit a constant fraction of the total number of delay times in the T-MSD with a minimum of three^2–5^. When the trajectories are short, even with a short fit range, the estimation of the diffusion constant can be biased due to correlation in the squared displacements.


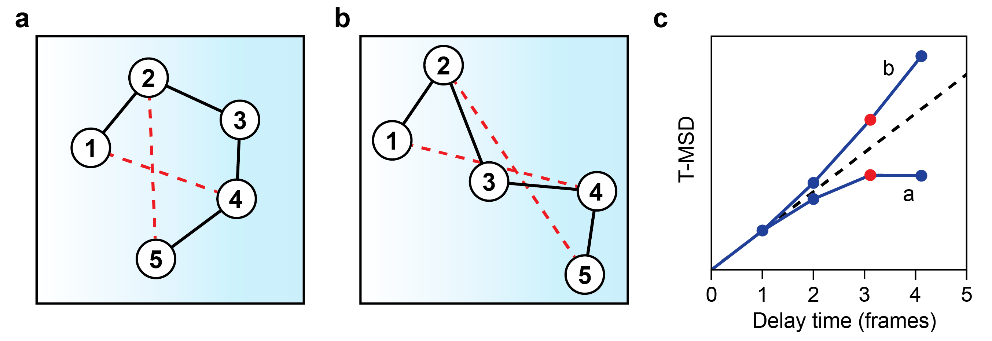


**Figure S1** a–b) Schematic spatial representation of two trajectories spanning five frames that could have the same underlying diffusion constant. The localizations are indicated by circles with the frame number noted inside. The black lines are guides to eye for visualization of the trajectories. The longest displacements are statistically dependent and highly correlated due to the shape of the trajectory, e.g., when the trajectory is folded back onto itself (a) or stretched out (b). This is illustrated by the similar length of the displacements with a delay time $t_{n}$ = 3 frames (red dashed lines). c) Schematic of the time-average mean squared displacement (T-MSD) curve of a single trajectory similar to those in (a–b). When the trajectory is folded onto itself (a), the longest displacements are shorter than expected for normal diffusion (black dashed line), while they are longer for a stretched trajectory (b). Correlations in the trajectory manifest itself in correlations in the T-MSD curve, which results in bias in the fitted parameters.

## Trajectory Simulation

We simulated synthetic trajectories of emitters exhibiting transient trapping following the procedure outlined in Fig. S2. Estimation of the localisation error of an out-of-focus emitter is not trivial, particularly when the emitter moves more than roughly one pixel per frame thereby introducing an additional localisation error called motion blur. On top of that, the localisation algorithm fails to recognise the emitter when it is far out-of-focus^6^. To simulate a set of trajectories that closely resembles an experimental data set, we generated realistic time-lapse movie frames from three-dimensional trajectory coordinates. We converted these time-lapse movies back in two-dimensional trajectories with third-party localization and tracking software—just like it would be done with experimentally obtained micrographs. In short, a synthetic dataset was generated in three steps: (1) simulation of the coordinates of 3-dimensional *xyzt* trajectories; (2–4) generation synthetic *xyt* time lapse video frames from 3-dimensional trajectories using a simulated point spread function; and (5) localisation and tracking of synthetic time-lapse video frames to obtain the simulated trajectory coordinates. Every trajectory coordinate in step 1 resulted in a PSF (step 2a) “placed” in the time-lapse video (step 2b). To simulate motion blur, the trajectory coordinates in step 1 were computed with a five times higher time resolution than the synthetic time lapse video in step 2b. This means that we placed five PSFs along the path the emitter has travelled during the frame. Photon-counting noise and camera noise were simulated in steps 2–4 resulting in an imprecision in the localization in (5), which altogether introduced a localization error. Each step is described in more detail below and the simulation parameters are given in Table S1.


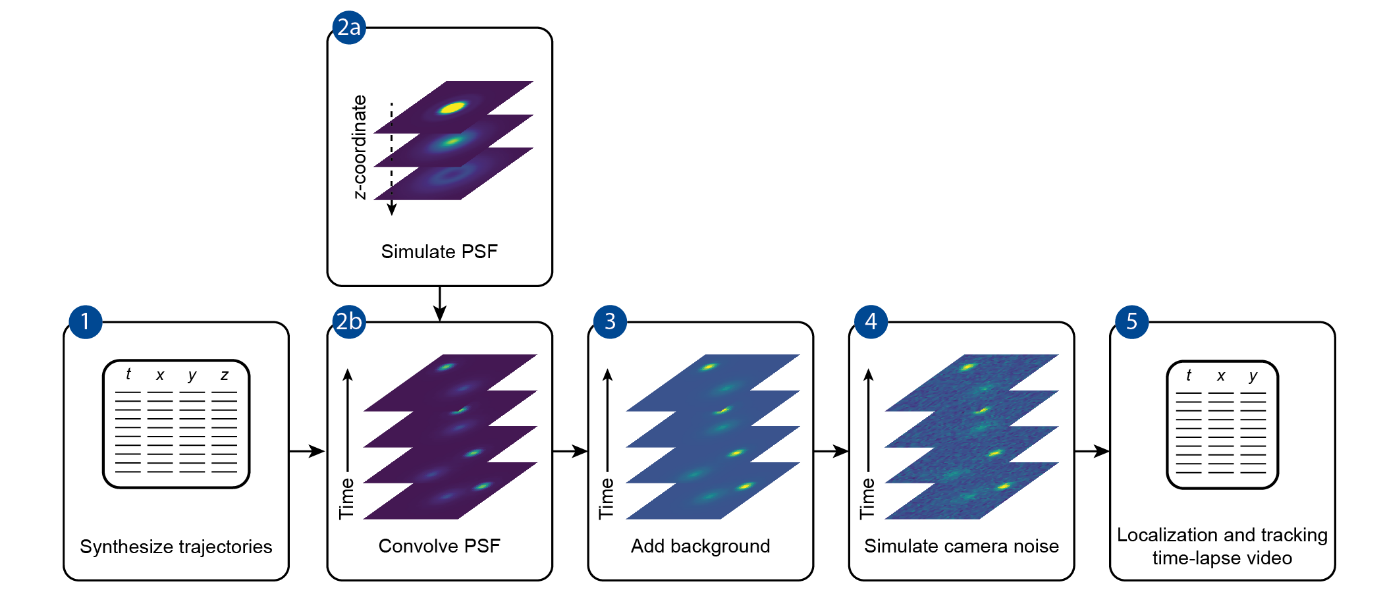


**Figure S2:** Schematic overview of the simulation of the synthetic dataset: (1) ‘raw’ trajectories are simulated and the *xyzt* coordinates are saved; (2) the PSF is simulated at various z-coordinates (2a), which is convolved with the trajectory coordinates (2b) to obtain a time-lapse video of the point emitters; (3) a fixed background is added; (4) camera noise is simulated over the signal in (3); and (5) the resulting time-lapse images are localized and tracked to obtain the simulated trajectories.

Three-dimensional trajectories were simulated as a multistate normal random walk (step 1, Fig. S2). The displacement probability in all directions was given by a normal distribution with a standard deviation of $\sqrt{2D_{i}t}$, with $D_{i}$ the diffusion coefficient of state $i$ and $t$ the delay time^7^. The diffusion state during one frame was computed from a discrete-time Markov chain with a specified state transition matrix and random initial state. This kind of model generates a sequence of events (diffusion states), where the probability of each event depends only on the state in the previous one. To simulate transient trapping, the diffusion constant of the first diffusion state was set to zero ($D_{1} = 0$) while the other was set to $D_{2}=1.0\times{10}^{-12}$ m^2^ s^-1^. The probability to change diffusion state was 0.02 per frame. Periodic boundary conditions were applied with a bounding box equal to the field of view in *x*, *y*, and 8.2 µm in *z*. The concentration of emitters was kept constant at a density of 0.034 µm^-3^ (5.66 × 10^-14^ M), which was achieved by simulation of a fixed number of trajectories for the full simulation time. The number of localisations during one frame was oversampled five times to simulate motion blur. The obtained synthetic trajectories are called the “raw” trajectories.

The raw trajectories were converted into time-lapse videos (step 2, Fig. S2). The coordinates of the trajectory were translated into images via the point spread function (PSF), which is the response of an optical system (microscope) to a point emitter. The measured PSF is dependent on the depth of the emitter with respect to the focus, and the recorded PSF changes as function emitter’s depth coordinates. We first simulated an isotropic PSF following vectorial diffraction theory as described by Backer and Moerner (step 2a, Fig. S2)^8^. The centre of the image of the PSF corresponding to the *z*-coordinates of the raw trajectory was convolved with the trajectory’s *x*,*y*-coordinates (step 2b, Fig. S2). Since we can only convolve the PSF image with whole pixels, we translated the PSF image before convolution to achieve subpixel resolution. The PSF was oversampled in *x*,*y* with respect to the pixel size of the time-lapse movie to ensure accurate interpolation during translation of the PSF image. To speed up the calculation, the *x*,*y*-coordinates of the trajectory were rounded to the nearest value on a grid with intervals of 0.1 pixel size. The normalized intensity of the in-focus PSF image was scaled by the photon flux per emitter. Altogether, this resulted in a dimensionality reduction, because the depth information in the time-lapse images is encoded in the shape of the PSF.

A constant auto-fluorescent background was added to the noise-free time-lapse images, which was subjected to an electron-multiplying charge-coupled device (EMCCD) camera noise model as described in Sage *et al.* (step 3–4, Fig. S2)^9^. We have taken the same model parameters because these values are similar for EMCCD cameras from other manufacturers. Finally, the simulated time-lapse images have been localized and tracked in DoM v.1.2.0^10^, an plugin for ImageJ, which we call the simulated trajectories (step 5, Fig. S2). We have excluded false positives in the tracking. The DoM results table was exported to .csv and imported in DiffusionLab.

**Table S1:** overview of the parameters used in the simulation of the synthetic dataset.

| **Parameter** | **Value** |
| --- | --- |
| **Experiment** |  |
| Field of view | 32.8$\times$32.8 µm^2^ |
| Pixel size | 64 nm |
| Number of pixels | 512$\times$512 |
| Number of frames | 2000 |
| Frame time | 50 ms |
| Temporal oversampling | 5 |
| Bounding box ($x\times y\times z$) | 32.8$\times$32.8$\times$8.2 µm^3^ |
| Number of emitters | 300 |
| Emitter density | 0.034 µm^-3^ |
| Diffusion constants [$D_{1};$ $D_{2}]$ | [0; 1$\times$10^-12^ ] m^2^ s^-1^ |
| Transition matrix | $\left( \begin{matrix} 0.98 & 0.02 \\ 0.02 & 0.98 \end{matrix} \right)$ frame^-1^ |
| Background | 100 s^-1^ pixel^-1^ |
| **Point spread function** |  |
| Number of photons per emitter | 12889 s^-1^ |
| PSF sampling $x,y$ | 10 nm |
| PSF sampling $z$ | 10 nm |
| Convolution resolution | 1 nm |
| Cut-off in $z$ | 1 µm |
| Wavelength | 600 nm |
| Refractive index | 1.518 |
| Magnification microscope | 91 |
| Numerical aperture | 1.40 |
| **Camera** |  |
| Quantum efficiency | 0.9 |
| Readout noise | 74.4 |
| Spurious charge | 0.0002 |
| Electron magnification gain | 300 |
| Baseline | 100 |
| Electrons per ADC | 45 |
| **Localization and tracking** |  |
| Sigma | 2.0 |
| Signal-to-noise | 3.0 |
| Blinking gap | 2 frames |
| Pixel jump | 20 pixels (training set: 15 and 20 pixels) |

## Find Important Properties for Manual Classification with the Biplot Tool

Sometimes it is hard to rationalise which properties are important for classification. DiffusionLab provides a tool to find these properties. This facilitates manual construction of the classification tree and allows easy modification and extension of existing trees. This tool is called a biplot and finds its origin in principal component analysis (PCA). The goal of PCA is to reduce the number of properties with the smallest loss of variance (‘information’) by making linear combinations of the input properties. These new properties are called principal components and are numbered from low to high. To find the first principal component, the variance described by this component is maximized. The second principal component has to be orthogonal to the previous, and again the information described by this component is maximized within this constraint. This process is repeated until the same number of principal components are found as input properties. Thus, the lowest principal components contain most information, while the highest do not contain much extra information and can be discarded to reduce the number of properties with a minimal loss of information.

The relation between the principal components, trajectories, and input properties can be visualized with a biplot. Fig. S3 shows the biplot at two splits in the classification tree example from the main text. Properties that have a large projection onto principal component 1 contain most information and are therefore interesting candidates for the next split. Indeed, the properties that have been found for the second and third split by machine learning contain much information. We find that the entropy could be a good descriptor instead of the MBCC–CoM at the second split. Similarly, the number of points contains much information at the third split, but also the length and minimum bounding circle (MBC) radius would be good descriptors here. This example shows that the biplot is a valuable tool to visualize and find good properties for classification, even when the classification tree is not known.


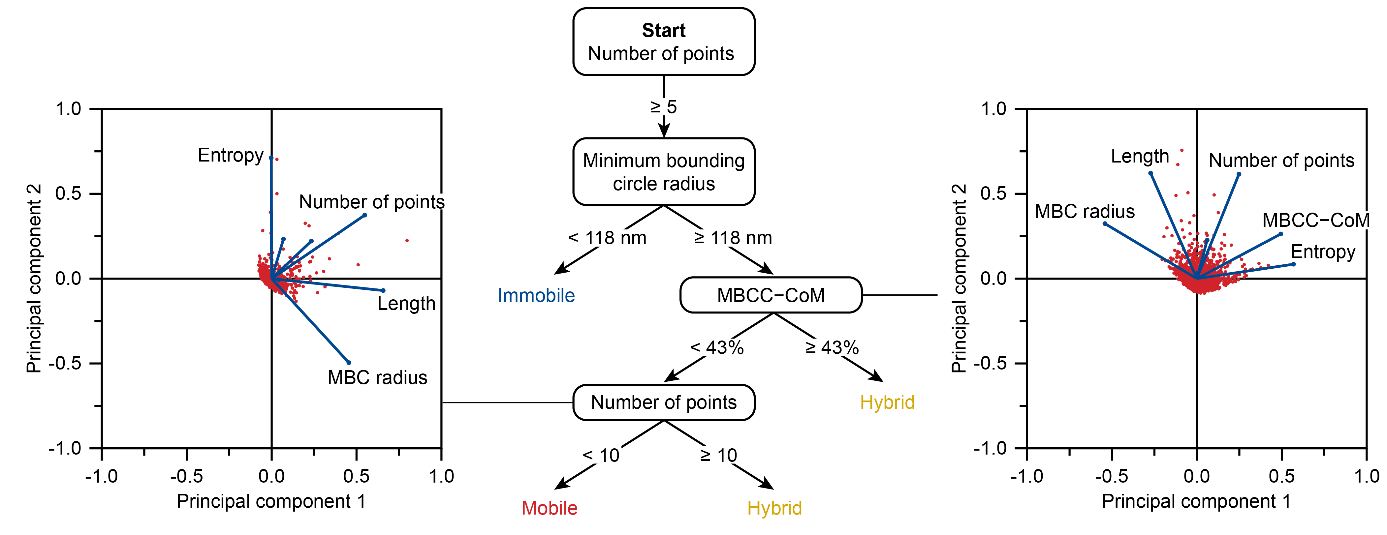


**Figure S3** Classification tree obtained with machine learning on a training set of 100 manually classified trajectories for a simulated dataset with transient trapping. The classification tree is read from top to bottom for each trajectory, weighing one property at each branch, until the track is classified as either mobile, immobile, or hybrid. A biplot of all properties (except the elongation angle and diffusion constant) at the second and third split is shown next to the classification tree. The biplot is a combination of the score plot (blue lines) and loading plot (red dots) as computed with principal component analysis. The blue lines (vectors) indicate the properties, with their direction being the contribution to each of the first two principal components and their length the magnitude of the contribution. Properties that are strongly correlated have their vectors in the same direction. Each trajectory is shown as red dot in principal component space.

## Trajectory Plot and Artifact Handling

In Fig. S4, we show the simulated trajectories prior to and after classification by the hierarchical classification tree in Fig. 2b. Classification appeared to be successful in most cases: mobile trajectories only have mobile displacements resulting in short trajectories; hybrid trajectories have both mobile and immobile displacements; and immobile trajectories have many immobile displacements distributed around a central spot. For an extensive discussion of the classification results, the reader is referred to Fig. 3 and the main text. The simulated trajectories are qualitatively similar to ones shown in Hendriks and Meirer et al.^11^ and Fu and Maris et al.^12^.

Some of the simulated trajectories are the result of “artifacts” or “mistakes” during the tracking, where it is clear that several trajectories belong to the same fluorophore and should have been linked together into one trajectory, or when the trajectories represent unphysical motion. In our experience, these artifacts are difficult to avoid completely and easily arise when many fluorophores are trapped or confined. We show how our classification tree handles these artifacts. In Fig. 4c, we find an immobile trajectory that overlaps with the immobile section of two hybrid trajectories (arrow 1). Due to missing localizations as a result of a low signal (or blinking of the fluorophore—not included in the simulation), long trajectories can be split into multiple shorter ones. The classification tree correctly identifies the individual components: a hybrid trajectory, followed by an immobile trajectory and a hybrid trajectory. In Fig. 4d, another type of artifact is shown. A crossover between two immobile fluorophores (arrows 2 & 3) occurs in a single trajectory. These artifacts are classified as hybrid, because they are similar to the naturally occurring hybrid trajectories in the data set, containing both mobile and immobile displacements. If these artifacts would occur frequently, they could have been classified into a separate population in the training set and removed from the analysis.


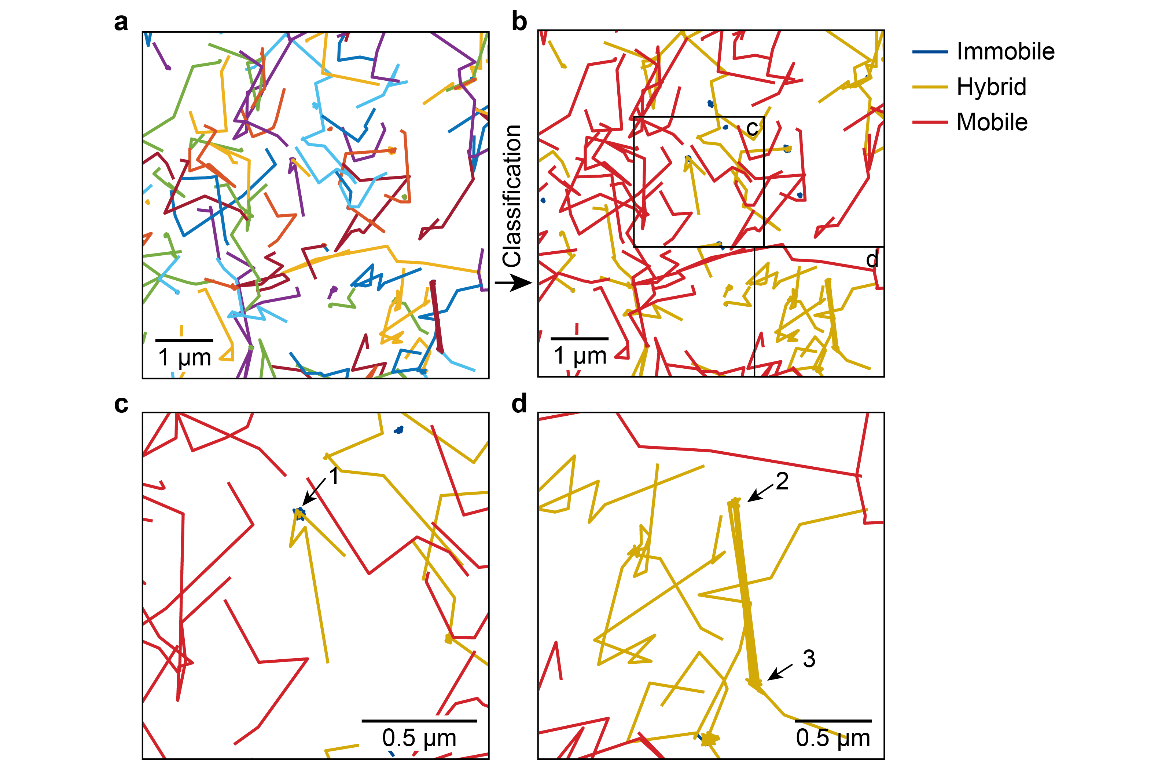


**Figure S4** a–b) Individual trajectories (≥5 localizations) prior to (a) and after classification (b) into immobile, hybrid, and mobile populations. Classification appears to be successful in most cases. c–d) Zoom-in of the trajectories in (b), where the numbered arrows indicate regions discussed in the text.

## Performance of Classification Models

We compared the accuracy of various classification models fitted to the training set used in the main text. We calculated two different metrics for the classification accuracy, which are given in Table S2. First, the accuracy without model validation was computed, that is one minus the resubstitution loss. Second, we computed the accuracy with validation using 20-fold cross validation. The model was trained with 95/100 trajectories and validated against the remaining 5/100, which was repeated until all trajectories had been part of the validation set, i.e., a 20-fold repetition. The accuracy computed with validation was slightly lower than the accuracy without validation. This is likely because the training set contained 5% fewer trajectories, which is significant for a small training set with only 100 trajectories, and resulted in a poorer performing model.

The classification tree model used in the main text performed well compared to the other tested models. Using the 20-fold cross validation accuracy metric, classification tree is even the best performing model. It is important to realize that the accuracy is computed against the user-generated training set that might not be perfectly representing the ground truth due to inconsequent classification by the user. An explanation for the good performance of the classification tree could be that the maximum number of splits is kept low (<10), which prevents overfitting and makes it less susceptible to imperfections in the training set. The reduction of the number of dimensions by principal component analysis before model fit drastically reduced the accuracy for this training set.

**Table S2** Accuracy of various models trained with the training set used for classification in the main text. The accuracy was computed via the resubstitution loss (no validation) and after 20-fold cross validation. The reported models are: the classification tree discussed in the main text; Gaussian Naive Bayes and Naive Bayes with a Gaussian Kernel; support-vector machines (SVM) with different kernel functions using an automatic kernel scale mode; K-nearest neighbours (KNN) with a default of 10 neighbours computed following various distance metrics (Euclidean, cosine, and cubic Minkowski). The medium and coarse KNN have respectively 1 and 10 nearest neighbours. The weighed KNN uses an Euclidean distance metric with the squared inverse distance weight. The classification tree was computed directly in DiffusionLab’s Classification Trainer App. The other models were computed in MATLAB’s Classification Learner App. Export of training sets to and import of classification models from MATLAB’s Classification Learner App are supported by DiffusionLab.

| **Model** | **Accuracy no validation (%)** | **Accuracy 20-fold cross validation (%)** |
| --- | --- | --- |
| Classification tree — main text | 98 | 95 |
| Naive Bayes — Gaussian | 91 | 89 |
| Naive Bayes — Kernel | 95 | 91 |
| SVM — Linear | 92 | 87 |
| SVM — Quadratic | 97 | 87 |
| SVM — Cubic | 100 | 87 |
| SVM — Gaussian | 98 | 83 |
| KNN — Medium | 84 | 80 |
| KNN — Coarse | 38 | 38 |
| KNN — Cosine | 86 | 81 |
| KNN — Cubic | 87 | 82 |
| KNN — Weighted | 100 | 84 |

## Robustness of Computation Classification Tree

The reproducibility of the classification tree computation is discussed in this section. Based on the principal component analysis, we find that there are a couple of equivalent trajectory properties for classification (Sec. S1.3). For instance, in the second split in the classification tree shown in Fig. S3, the MBCC–COM, entropy, and minimum bounding circle radius have a large projection on the first principal component. We expect that a similar training set to the one used in main text would result in a similar classification tree, but based on different ‘equivalent’ trajectory properties. Interestingly, we generally find variations on the same classification tree with the minimum bounding circle radius at the first split and the MBCC–COM at the second split. Moreover, the number points is frequently after either the MBCC–COM branches. An example is shown in Fig. S5a and the analysis of all classified trajectories is shown in Fig. S5b–g, which was plotted on the same axes as in Fig. 3a–f for comparison. The similarity both in classification tree and motion analysis results is striking. The classification tree has a resubstitution loss of 6%, which is 4% higher than for the classification tree in the main text. The minimum bounding circle radius is smaller in the classification tree presented in this section (67 nm vs. 118 nm), which is compensated for by an extra split based on the minimum bounding circle radius to separate the mobile and immobile trajectories. Other splits are similar in both classification trees. As expected, the histogram of trajectory diffusion constants after classification (Figs. 3b and S5c), TE-MSD after classification (Figs. 3d and S5e), and one minus the cumulative distribution function (CDF) of squared displacements (Figs. 3f and S5g) are almost identical. The obtained diffusion constant of the TE-MSD fit of the mobile population is slightly lower than for the data set shown in the main text (*D* = 8.98 ± 0.15 $\times$ 10^-13^ m^2^ s^-1^ vs. 9.23 ± 0.14 $\times$ 10^-13^ m^2^ s^-1^), which can be explained by the lightly higher fraction of immobile displacements in the 1 – CDF (5–6% vs. 4%). Altogether, the classification into mobile motion behaviour is marginally better in the classification tree shown in the main text.


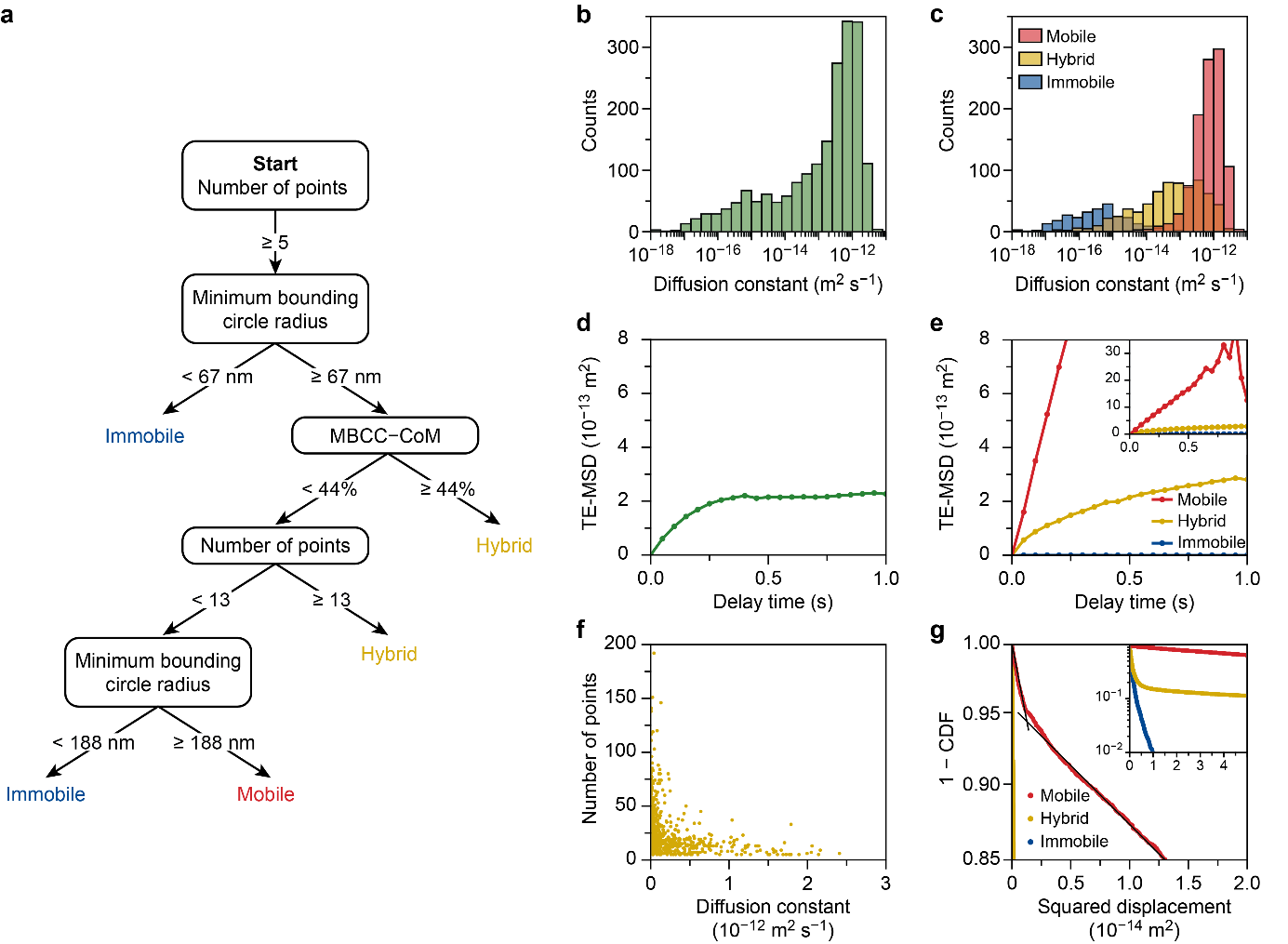


**Figure S4** a) Classification tree obtained with machine learning on a training set of 100 manually classified trajectories for a simulated dataset with transient trapping. The training set comprises a different subset of classified trajectories than shown in the main text in Figs. 2–3. A maximum number of 5 splits was set for this tree, and the full tree (without maximum number of splits) yielded virtually the same results. b–c) Histogram of the measured diffusion constant as obtained per trajectory time-averaged mean squared displacement (T-MSD) analysis before b) and after classification c). Negative measured diffusion constants are not displayed in the logarithmic scale. d–e) Zoom of the time–ensemble averaged mean squared displacement (TE-MSD) before d) and after classification e). A zoom-out is shown in the inset in e). f) Correlation plot of the diffusion constant and number of points of the hybrid population. Each dot represents a single trajectory. g) One minus the cumulative distribution function (CDF) of squared displacements for a delay time of one frame of all trajectories plotted with a logarithmic *y*-axis. The colours indicate the populations after classification. The solid black lines are a guide to the eye to indicate two regimes in the cumulative density probability of the mobile trajectories. A zoom-out is shown in the inset.

## Probability of an Immobile Trajectory to Fall Within the Minimum Bounding Circle Radius

We consider how likely immobile trajectories fall entirely within a minimum bounding circle with some threshold radius. If an immobile emitter is localised over many images, the localisation coordinate in one dimension would be normally distributed around the true position of the emitter. The localisation error *σ* is defined as the standard deviation of this normal distribution. Thus, assuming that the centre of the minimum bounding circle is equal to the true position of the emitter, we can compute the fraction of localisations that are measured within the threshold radius $\alpha$

$\varepsilon(\text{α}\text{,}\sigma)=\mathrm{erf} \left( \frac{\text{α}}{\sigma\sqrt{2}} \right)$ (Eq. S1)

with $\mathrm{erf}$ the error function. Then, the probability that all localizations of a trajectory fall within the minimum bounding circle with threshold radius $\alpha$ is simply

$\gamma=\varepsilon^{k}$ (Eq. S2)

with $k$ the number of localisations in a trajectory. This value is reported in the main text for $k$ = 17, which is the mean number of localisations for the immobile population, and this value is also used for the computations reported below.

First, to compute $\varepsilon$ and $\gamma$, we need to obtain a value for the localisation error $\sigma$. We estimate *σ* as the best possible localisation uncertainty obtained with a least-squares fit of a two-dimensional Gaussian distribution to the observed point spread function. This limit was first described by Thomson et al.^13^ and later corrected by Mortensen et al.^14^ and is

$\sigma_{g}^{2}=\frac{\sigma_{a}^{2}}{N}\left( \frac{16}{9}+\frac{8\pi\sigma_{a}^{2}b^{2}}{Na^{2}} \right)$ (Eq. S3)

with $\sigma_{a}^{2}=\sigma_{\mathrm{PSF}}^{2}+{a^{2}}/{12}$, $\sigma_{PSF}$ the standard deviation of the point spread function,$a^{2}$ the pixel area, $N$ the signal photon count, and $b$ the background photons per pixel. Eq. S3 is multiplied by a factor two to account for the excess noise introduced by the electron multiplication process in the EMCCD camera. The formula reproduces the localisation error found in computer experiments with a known truth^14^. To estimate the localization error of an out-of-focus emitter, we compute $\sigma_{\mathrm{PSF}}$ following Deschout *et al.* for an emitter with a distance $z$ from the focal plane^15^

$\sigma_{\mathrm{PSF}}^{2}(z)=\sigma_{\mathrm{PSF},0}^{2}\left( 1+\frac{z^{2}}{\left[ \left( 4\pi n/\lambda\right){\sigma_{PSF,0}}^{2} \right]^{2}} \right)$ (Eq. S4)

with $\sigma_{PSF,0}$ the standard deviation of the point spread function at the in-focus plane, $n$ the refractive index of the immersion medium, and $\lambda$ the emission wavelength. The $\sigma_{\mathrm{PSF},0}$ was extracted from a fit of the true (simulated) point spread function at the in-focus plane and was found to be 98.6 nm. The values for $a^{2}$, $N$, $b$, $n$, and $\lambda$ were taken from Table S1, with $N$ the number of photons per emitter multiplied by the camera quantum efficiency. When in focus, $z$ = 0, Eq. S4 reduces to $\sigma_{\mathrm{PSF}}^{2}(0)=\sigma_{\mathrm{PSF},0}^{2}$ resulting in $\sigma_{g}$ = 12.4 nm, which was substituted for $\sigma$ in Eq. S1 and S2. For a minimum bounding circle with threshold radius $\alpha=$ 118 nm, the probability to find a single localization within this circle is $\varepsilon$ > 99.99% and the probability to find a trajectory is $\gamma$ > 99.99%. The probability that all localizations of a trajectory fall a minimum bounding circle with threshold radius $\alpha=$ 118 nm is still virtually one when the emitter moves out of focus. For an emitter with $z$ = ±400 nm, we find $\sigma_{g}$ = 28.2 nm, ε > 99.99%, and $\gamma$ = 99.95%.

## Classification of Transient Confinement with a Modified Classification Tree

We demonstrate how the reported classification tree (Fig. 2b) can be adapted for segmentation of trajectories with qualitatively similar motion behaviour. Instead of trapping with *D*_1_ = 0 m^2^ s^-1^, we simulated confinement parallel to the observation plane in a circular disk with a radius of 500 nm using reflective boundary conditions^16^. The trajectories were simulated with the same parameters as in the transient confinement example discussed in the main text. The adapted classification tree and the individual trajectories at each classification end point branch is shown in Fig. S5. We manually changed the value of the minimum bounding circle radius (MBCR) at the first split until all trajectories that appeared fully confined were captured. The optimum value of 550 nm just includes the mode of the MBCR histogram (Fig. S5a). This indicates that trajectories which explore an area corresponding to the confinement domain are most abundant (Fig. S5b, arrow 1). A number of short trajectories are also included in the confined population; however, their classification is ambiguous as they only explore a limited area (Fig. S5b, arrow 2). The user could decide to group these trajectories with the mobile trajectories. This can be done by adding an additional split at the “confined” branch, segmenting based on the number of points. At the split of the minimum bounding circle centre minus the centre of mass (MBCC–CoM), the distribution in the histogram is not bimodal as in the classification tree for transient adsorption (Fig. 2b). Nevertheless, we find that approximately the same threshold as used in Fig. 2b filters out hybrid trajectories with a mobile segment connected to confined segment (Fig. S5a and Fig. S5b, arrow 3). In the next split, we increased the threshold of the number of points to 18 (Fig. S5a), which makes a good distinction between mobile trajectories and hybrid trajectories with multiple domains of confinement or two mobile segments connected with an immobile one (Fig. S5b, arrows 4 & 5).


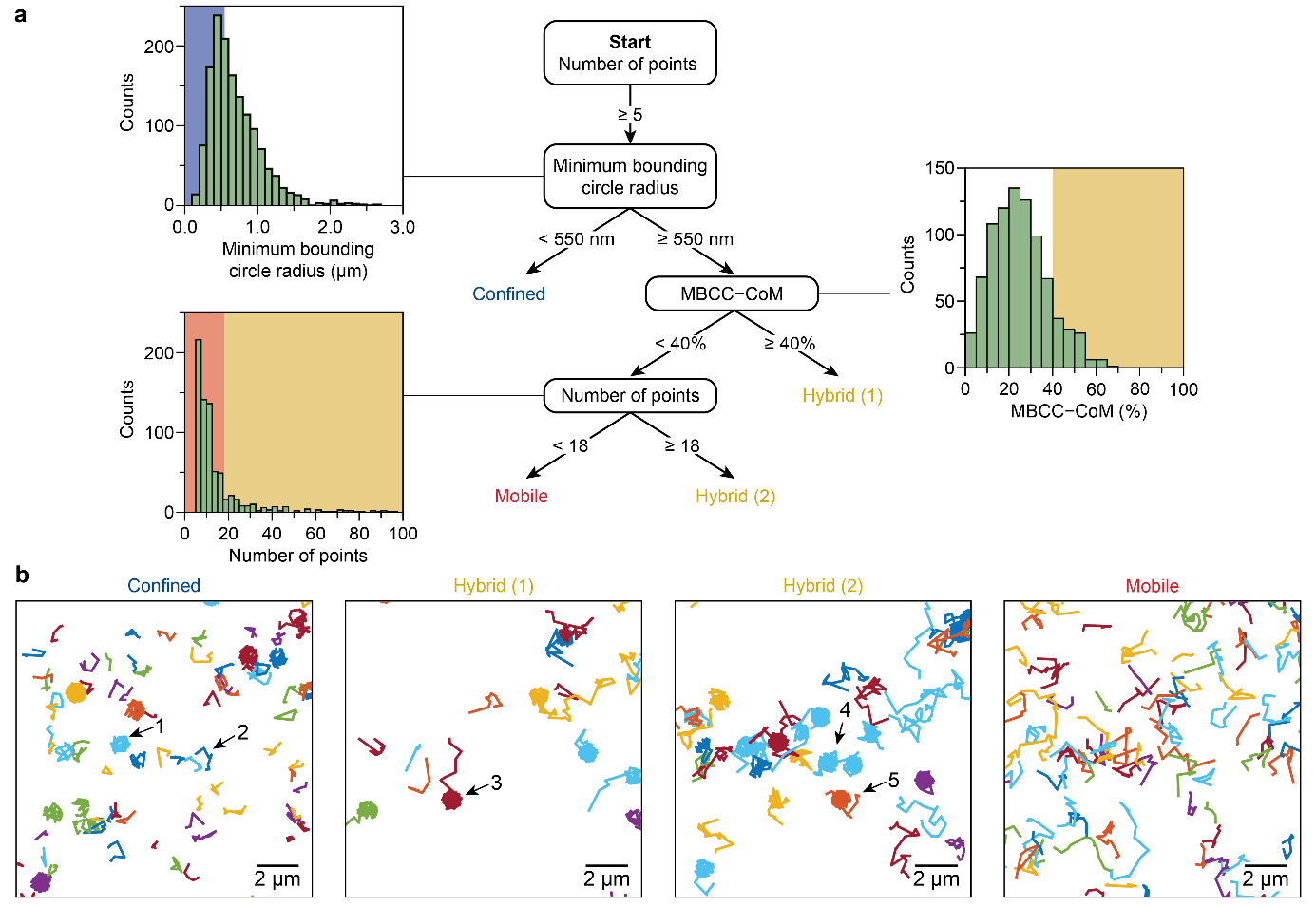


**Figure S5** a) Classification tree obtained by manual modification of the thresholds in the classification tree shown in Fig. 2b for a simulated dataset with transient confinement. The classification tree is read from top to bottom for each trajectory, weighing one property at each branch, until the trajectory is classified as either confined, hybrid or immobile. At each split, the histogram of the property is given for all trajectories of the branch, with the background colour indicating the threshold value of the split. b) Individual trajectories of the same region of interest at each end point branch: confined, hybrid (1), hybrid (2), and mobile. The numbered arrows indicate regions discussed in the text.

We show the motion characteristics of the classified populations via the time–ensemble averaged mean squared displacement (TE-MSD, Fig. S6a). We find that the diffusion constant of the mobile trajectories is *D* = 1.099 ± 0.016 $\times$ 10^−12^ m^2^ s^-1^, which is approximately 10% larger than simulated value of 1 $\times$ 10^−12^ m^2^ s^-1^. The origin of the overestimation of the diffusion constant is a result of the relatively large MBCR of 550 nm. As discussed in the previous paragraph, the classification of the trajectories that fall within the MBCR is ambiguous. Short trajectories with short displacements could have been selectively filtered out at this split, resulting in an overestimation of the diffusion constant. Indeed, the addition of an extra split removing mobile trajectories from the confined population as described before, using a ‘number of points < 7’, resulted in a more accurate estimation of the diffusion constant of the mobile trajectories, i.e., *D* = 1.011 ± 0.006 $\times$ 10^−12^ m^2^ s^-1^. This demonstrates that the adaptation of a classification tree to a qualitatively similar data set in some cases requires the addition or removal of splits. We fitted the TE-MSD of the confined population with a model describing confinement on a circular disk (Fig. S6b). The mean squared displacement (MSD) can be described by

$\mathrm{MSD}= R^{2}\left( 1-\exp\left( -\frac{t_{n}}{\tau} \right) \right)$ (Eq. S5)

with *t_n_* the delay time, *R* the radius of the circular disk, and *τ* the characteristic equilibration time after which the effect of the confinement boundaries become evident and the MSD fully flattens^4,7,17,18^. We obtained *R* = 475 ± 28 μm and correctly retrieved the simulated confinement radius of 500 nm^17^.


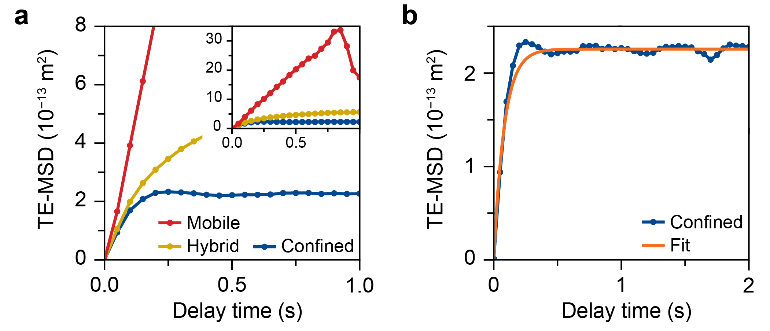


**Figure S6** a) Zoom of the time–ensemble averaged mean squared displacement (TE-MSD) of the transient confinement data set after classification. A zoom-out is shown in the inset. b) Fit of the confined population in a) with Eq. S5.

# References

1. Vestergaard, C. L., Blainey, P. C. & Flyvbjerg, H. Optimal estimation of diffusion coefficients from single-particle trajectories. *Phys. Rev. E* **89**, 022726 (2014).

2. Michalet, X. Mean square displacement analysis of single-particle trajectories with localization error: Brownian motion in an isotropic medium. *Phys. Rev. E* **82**, 041914 (2010).

3. Michalet, X. & Berglund, A. J. Optimal diffusion coefficient estimation in single-particle tracking. *Phys. Rev. E* **85**, 061916 (2012).

4. Qian, H., Sheetz, M. P. & Elson, E. L. Single particle tracking. Analysis of diffusion and flow in two-dimensional systems. *Biophys. J.* **60**, 910–921 (1991).

5. Kepten, E., Weron, A., Sikora, G., Burnecki, K. & Garini, Y. Guidelines for the Fitting of Anomalous Diffusion Mean Square Displacement Graphs from Single Particle Tracking Experiments. *PLOS ONE* **10**, e0117722 (2015).

6. Vestergaard, C. L. Optimizing experimental parameters for tracking of diffusing particles. *Phys. Rev. E* **94**, 022401 (2016).

7. Saxton, M. J. Modeling 2D and 3D Diffusion. in *Methods in Membrane Lipids* (ed. Dopico, A. M.) 295–321 (Humana Press, Totawa, 2007).

8. Backer, A. S. & Moerner, W. E. Extending Single-Molecule Microscopy Using Optical Fourier Processing. *J. Phys. Chem. B* **118**, 8313–8329 (2014).

9. Sage, D. *et al.* Super-resolution fight club: assessment of 2D and 3D single-molecule localization microscopy software. *Nat. Methods* **16**, 387–395 (2019).

10. Katrukha, E. A., Cloin, B., Teeuw, J. & Kapitein, L. C. Detection of Molecules plugin for ImageJ, Utrecht University. https://github.com/ekatrukha/DoM_Utrecht (2017).

11. Hendriks, F. C. *et al.* Single-Molecule Fluorescence Microscopy Reveals Local Diffusion Coefficients in the Pore Network of an Individual Catalyst Particle. *J. Am. Chem. Soc.* **139**, 13632–13635 (2017).

12. Fu, D. *et al.* Unravelling Channel Structure–Diffusivity Relationships in Zeolite ZSM-5 at the Single-Molecule Level. *Angew. Chem. Int. Ed.* **64**, e202114388 (2022).

13. Thompson, R. E., Larson, D. R. & Webb, W. W. Precise Nanometer Localization Analysis for Individual Fluorescent Probes. *Biophys. J.* **82**, 2775–2783 (2002).

14. Mortensen, K. I., Churchman, L. S., Spudich, J. A. & Flyvbjerg, H. Optimized localization analysis for single-molecule tracking and super-resolution microscopy. *Nat. Methods* **7**, 377–381 (2010).

15. Deschout, H., Neyts, K. & Braeckmans, K. The influence of movement on the localization precision of sub-resolution particles in fluorescence microscopy. *J. Biophotonics* **5**, 97–109 (2012).

16. Volpe, G., Gigan, S. & Volpe, G. Simulation of the active Brownian motion of a microswimmer. *Am. J. Phys.* **82**, 659–664 (2014).

17. Mortensen, K. I., Flyvbjerg, H. & Pedersen, J. N. Confined Brownian Motion Tracked With Motion Blur: Estimating Diffusion Coefficient and Size of Confining Space. *Front. Phys.* **8**, (2021).

18. Manzo, C. & Garcia-Parajo, M. F. A review of progress in single particle tracking: from methods to biophysical insights. *Rep. Prog. Phys.* **78**, 124601 (2015).
